# Histamine and migraine revisited: mechanisms and possible drug targets

**DOI:** 10.1186/s10194-019-0984-1

**Published:** 2019-03-25

**Authors:** Jacob Worm, Katrine Falkenberg, Jes Olesen

**Affiliations:** Danish Headache Center and Department of Neurology N39, University of Copenhagen, Rigshospitalet Glostrup, DK-2600 Copenhagen, Denmark

**Keywords:** Migraine, Histamine, Antihistamines, Histamine receptors, Drug targets

## Abstract

**Objective:**

To review the existing literature on histamine and migraine with a focus on the molecule, its receptors, its use in inducing migraine, and antihistamines in the treatment of migraine.

**Background:**

Histamine has been known to cause a vascular type headache for almost a hundred years. Research has focused on antihistamines as a possible treatment and histamine as a migraine provoking agent but there has been little interest in this field for the last 25 years. In recent years two additional histamine (H_3_ and H_4_) receptors have been discovered and a series of non-sedating antihistamines have been developed. It is therefore timely to review the field again.

**Methods:**

For this review the PubMed/MEDLINE database was searched for eligible studies. We searched carefully for all articles on histamine, antihistamines and histamine receptors in relation to migraine and the nervous system. The following search terms were used: histamine, migraine disorders, migraine, headache, antihistamines, histamine antagonists, clinical trials, induced headache, histamine H_3_ receptor, histamine H_4_ receptor and pharmacology. Four hundred thirty-six titles were read, 135 abstracts were read, 112 articles were read in full and 53 articles were used in this review. Review process resulted in 12 articles added to a total of 65.

**Findings:**

Early studies of H_1_ and H_2_ antihistamines lack scientific strength and show conflicting results. Most of the antihistaminic drugs used in these trials bind also to other receptors which makes it difficult to conclude on the antihistaminic effect. Histamine is an efficient inducer of migraine attacks in migraine patients by an H_1_ mechanism most likely extracerebrally. These findings merit further investigation of antihistamines in clinical drug trials. The H_3_ and H_4_ receptors are found in primarily in CNS and immune tissues, respectively. H_3_ is likely to be involved in antinociception and has been linked with cognitive, neurodegenerative and sleep disorders. The only marketed H_3_ agent, pitolisant, is a brain penetrant H_3_ antagonist/inverse agonist which increases central histamine and causes headache. The experimental H_3_ agonist N^α^-methylhistamine has shown promising results as a migraine preventative in studies of uncertain quality. With the current limited knowledge of the H_4_ receptor it is questionable whether or not the receptor is involved in migraine.

**Conclusion:**

There is insufficient support for first generation antihistamines (both H_1_ and H_2_) as preventive migraine medications and sedation and weight gain are unacceptable side effects. Non-sedating H_1_ antihistamines need to be appropriately tested. Central H_3_ receptors seem to have a role in migraine that merit further investigation. The histaminergic system may be a goal for novel migraine drugs.

## Introduction

Histamine is a biogenic monoamine synthesized from the amino acid L-histidine by the L-histidine decarboxylase and metabolized by the enzyme histamine N-methyltransferase, using S-adenosylmethionine as a methyl donor. Histamine is located throughout the entire organism with high concentrations in lungs, skin and gastrointestinal tract. It is synthesized and stored in mast cells and basophils. It plays a role in multiple mechanisms both immunological and physiological, stimulating gastric secretion, inflammation, smooth muscle contraction, vasodilatation, permeability and much more. Histamine also functions as a neurotransmitter. It is synthesized in histaminergic neurons located in the posterior hypothalamus. These neurons have axons extending throughout the brain. Histamine carries out its effects via 4 subtypes of 7-transmembrane G-protein coupled receptors; H_1_, H_2_, H_3_ and H_4_ receptors [1–3].

The relation between histamine and headache has been on the agenda for almost a hundred years. A multitude of studies have been published focusing on the ability of histamine to induce headache and on the effect of antihistamines in the treatment of headache. During the last two decades the issue has, however, received almost no interest apart from two reviews [2, 4]. One reason that we now take this issue up is that there has been a massive development in the basic understanding of histamine biology, including the growing knowledge of histamine H_3_ and H_4_ receptors. Another is that a series of new generation antihistaminic drugs with or without the ability to penetrate the blood brain barrier have become available. It is therefore timely to review the issue.

## Methods

Literature used in this review was primarily searched in the PubMed/MEDLINE database and we limited our search to publications in English. References were selected by reading titles and abstracts judging relevance to the topic. We began our search with a screening to identify existing reviews of histamine and migraine. The search histamine [MeSH Terms] OR histamine [All Fields]) AND migraine disorders [MeSH Terms] OR migraine [All Fields] AND disorders [All Fields] OR migraine disorders [All Fields] OR migraine [All Fields] with only articles from the last 5 years resulted in 41 articles of which 9 abstracts were read. We then searched for the very early literature of histamine and vascular headaches. This literature dates back almost a century and a systematic search is not possible. Therefore, bibliography from the PhD thesis by Lassen LH was examined to obtain references. Six articles were found eligible. To find articles regarding the studies of histamine antagonists in migraine prophylaxis we searched for the terms: antihistamine AND (migraine OR headache) AND clinical trial from 1945 to 1985. This resulted in 68 articles of which 41 abstracts were read. Articles concerning migraine provocation studies with histamine were searched for using the terms: histamine AND induced AND headache from 1980 to present. One hundred twenty-three articles were found of which 24 abstracts were read. Articles of histamine H_3_ and H_4_ receptor pharmacology was searched for by the MeSH terms: Pharmacology AND Receptors, histamine with review as publication type. The search lead to 44 articles of which 21 abstracts were read. Individual free text searches of histamine H3 receptor OR histamine H4 receptor AND pharmacology from 2000 until present was made and yielded 160 articles of which 40 abstracts were read. In addition to the search results we looked through the reference lists of the selected articles to find more relevant studies. The search combined resulted in 436 titles, of which 135 abstracts were read and 112 articles were read in full. Fifty-three were included in this review. Review process resulted in 12 articles added to a total of 65.

### Historical overview

**Fig. 1:**
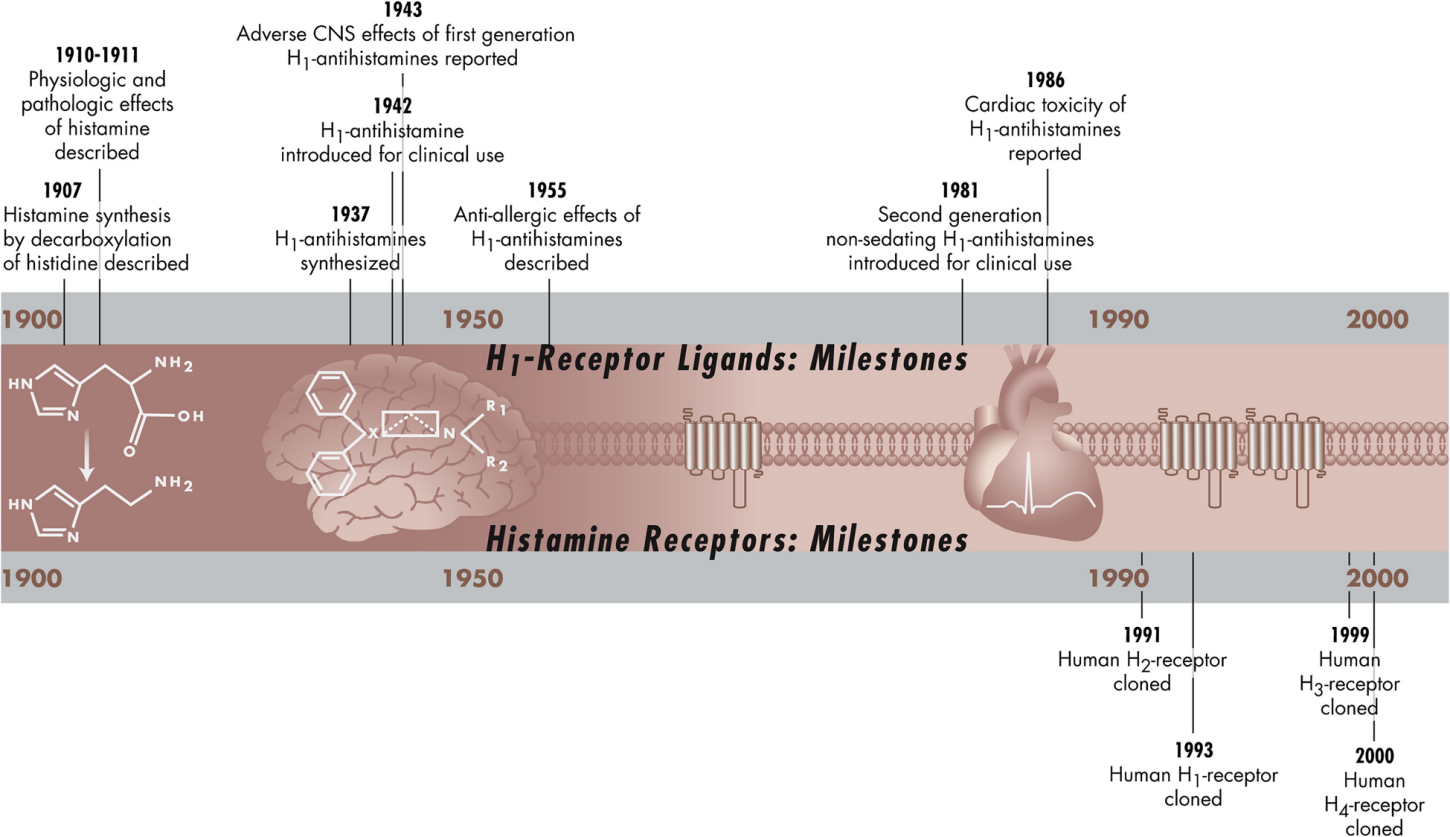
Historical timeline of histamine and its receptors. Reprinted from (11) Copyright© 2011 with permission from Elsevier, J Allergy Clin Immunol

The research of histamine began over a century ago [5], with the first antihistamines being introduced to clinical use around 70 years ago (Fig. 1) [1]. In 1926 Harmer and Harris were the first to describe headache after infusion of histamine [6]. In the 1930s Pickering did a series of careful experimental studies. Although none of them were placebo controlled, histamine was intelligently investigated for its ability to induce a vascular headache. The headache after infusion of histamine was thoroughly described and it was shown that histamine induced headache in a dose-dependent manner but that there was great variability between subjects. Pickering proposed that the origin of the headache was intracranial. He suggested stretching of large intracranial arteries to be a possible explanation of the perceived pain because headache severity was reduced by lowering blood pressure or raising intracranial pressure (i.e. less expansion of the vasculature). He concluded that it is in the dura mater, via its trigeminal nerve supply, that the headache arises [7].

In the following years several papers investigated the origin of histamine induced headache. Northfield injected histamine in the external and internal carotid artery and concluded that the pain arose from the internal carotid artery i.e. not from dura mater [8]. Another paper argued that the origin of pain in migraine headache is different from that of a histamine induced headache because of the absence of relief of migraine headache by increasing intracranial pressure while confirming a decrease of histamine induced headache [9]. Von Storch investigated the minimum dose of histamine required to induce headache. Migraineurs seemed to be more susceptible to histamine induced headache than control subjects [10]. Histamine was also tested as a treatment of migraine [11, 12]. The idea was to induce histamine tolerance in migraineurs based on a theory that these patients had lowered tolerance thus susceptible to external triggers of histamine (i.e. foods, antigens etc.). A method found successful but difficult to practice. The older literature had thus shown beyond doubt that histamine could induce a vascular type headache, the characteristics of which were carefully described. But how close was it to migraine? This could not be determined because there were no internationally accepted diagnostic criteria for migraine.

Experimental in vitro and animal studies have been performed adjacent to the clinical studies investigating histamines vasodilatory effect in the meningeal blood vessels as part of the underlying mechanism of migraine [13]. The research began decades ago with multiple studies published around the millennium [14] and researchers continue to investigate the endogenous mediators including histamine for their ability to generate migraine pain [15]. Histamine has been extensively studied alongside cytokines, chemokines and vasoactive peptides (i.e. the inflammatory soup) to gain knowledge of the underlying cellular mechanisms producing and maintaining the pain of migraine. At present a link between migraine and neurogenic inflammation comprising mast cell degranulation is widely accepted and this has been reviewed extensively most recently by Ramachandran [16].

### Are H_1_ and H_2_ blockers effective in migraine treatment?

Because histamine induces a vascular headache with similarity to migraine, it was logical to test antihistamines in the treatment of migraine when the class of antihistamines became available. These old drugs were sedative and soon became classified as histamine receptor 1 and 2 antagonists as no other histamine receptors or antagonists were known at the time. The literature on clinical trials of the H_1_ and H_2_ receptor antagonists in the prevention of migraine is rather scarce. Few papers have been published. Even fewer are randomized controlled trials and mostly used drugs that did not bind exclusively to H_1_ or H_2_ receptors. The following section examines the existing literature on antihistamine trials performed in the years from around 1960 to 2014 (see Table 1 for overview). All the existing literature describes antihistamines as histamine receptor antagonists. Today most of these drugs are defined pharmacologically as inverse agonists i.e. inhibitors of the basal receptor activity instead of being neutral antagonists which solely block the agonist response [3, 17]. For ease, we will describe all antihistamines as compounds with a histamine antagonistic effect.

**Table 1:** Antihistamines in migraine prophylaxis

Type (Ligand)	Participants	Daily dose	Duration	Main findings	First author (year)
H_1_ antagonist (cyproheptadine) vs. 5HT antagonist (methysergide) vs. Bellergal^b^ vs. placebo	453^a^	12–24 mg 6 mg NA NA	6 months	Clinical improvement (Headache free or substantial improvement)c: methysergide 64%, cyproheptadine 46%, Bellergal 34%, placebo 20%.	Curran (1964) (12)
Antihistaminic drug (pizotifen)	32^a^	1.5–2 mg	NA	Cessation or attenuation of attacks in 33%. Significant reduction in migraine days and increased response to acute medication in 40%. Increased resilience against induced attacks. AE^d^: weight gain and drowsiness.	Sicuteri (1967) (13)
Antihistaminic drug (pizotifen)	11 MA^e^ 27 MO^f^ 2 Cluster headache	1.5 mg (1–3 mg)	2–6 mo: 16 > 6 mo: 17	Significant (42.5%) clinical improvement compared to previous placebo reports (Curran 1964).	Selby (1970) (14)
H_1_ antagonist (cyproheptadine) vs. antihistaminic drug (pizotifen) vs. antihistaminic drug (methdilazine)	165^a^	12–24 mg 4.5–9 mg 16–32 mg	4 weeks	Clinical improvement: pizotifen 58%, methdilazine 41%, cyproheptadine 40% No placebo control.	Lance (1970) (15)
Antihistaminic drug (pizotifen) vs. 5HT antagonist (divascan) vs placebo	4 MA^e^ 26 MO^f^	3 mg 15 mg	8 weeks	Significant lowered attack rate with pizotifen. AE^d^: weight gain and drowsiness.	Osterman (1977) (16)
Antihistaminic drug (pizotifen) vs placebo	28^a^	3 mg	12 weeks	Complete resolution in 6 cases, reduced frequency and severity in 6 and no improvement in 2 cases. No improvement in the placebo group. AE^d^: weight gain and dizziness.	Lawrence (1977) (17)
H_2_ (cimetidine) vs. H_2_ and H_1_ combined (cimetidine and chlorpheniramine) vs. placebo	24 MO 1 basilar migraine	200 mg cimetidine 4 mg chlor pheniramine	1 week	No significant improvement over placebo with H_2_ antagonist alone or in combination with H_1_antagonist. AE^d^: weight gain and drowsiness.	Anthony (1978) (18)
H_2_ (cimetidine) vs. H_2_ and H_1_ combined (cimetidine and chlorpheniramine) vs. placebo	6 MA 28 MO	200 mg cimetidine 4 mg chlor-pheniramine	12 weeks	No significant improvement over placebo with H_2_ antagonist alone or in combination with H_1_antagonist. AE^d^: weight gain and drowsiness.	Nanda (1980) (20)
H_1_ (cinnarizine)	11MA^e^ 69 MO^f^	75 mg	14 weeks	Significant reduction in migraine days and use of acute medication. AE^d^: weight gain, drowsiness, mild reversible depression and dyspepsia. Open label. Dropout rate 3.75%.	Rossi (2003) (22)
H_1_ (cinnarizine)	60^g^	Up to 75 mg	12 weeks	Significant reduction in migraine days. AE^d^: Palpitations and dizziness. Open label. Dropout rate 5%.	Togha (2006) (21)
H_1_ (cinnarizine) vs valproate	86 MO^f^ 18 MA^e^	50 mg Cinnarizine 400 mg valproate	12 weeks	Valproate more effective than cinnarizine. AE^d^: Dry mouth, fatigue and somnolens as the most frequent. No placebo control. Dropout rate: 23% cinnarizine, 19.4% valproate.	Bostani (2013) (23)
H_1_ (cinnarizine) vs placebo	68 children (5-17y)^a^	50 mg or 1.5 mg/kg(> 30 kg)	12 weeks	Significant better than placebo to reduce headache frequency by at least 50% reduction. AE^d^: weight gain and drowsiness. Dropout rate: 12% cinnarizine, 5% placebo.	Ashrafi (2014) (24)

^a^No subtype stated

^b^Mixture of phenobarbitone 20 mg, ergotamine tartrate 0.3 mg and belladonna alkaloids 0.1 mg

^c^Not placebo corrected

^d^Adverse effects

^e^Migraine with aura

^f^Migraine without aura

^g^Subtype unknown

In a placebo controlled, but not double blinded or randomized study, Curran and Lance investigated cyproheptadine, an antiserotonin agent with H_1_ receptor antagonism, methysergide, a serotonin antagonist and bellergal (mixture of phenobarbitone 20 mg, ergotamine tartrate 0.3 mg and belladonna alkaloids 0.1 mg). Their ability to reduce attack frequency was compared and clinical improvement was defined as either headache free or substantially improved. Ninety-two patients received 12–24 mg/day of cyproheptadine, 137 patients received 6 mg/day of methysergide, 174 patients received the bellergal mixture and 50 were treated with placebo. Clinical improvement was 34% with bellergal, 64% with methysergide, 46% with cyproheptadine and 20% with placebo. No statistical analysis was made comparing cyproheptadine with placebo [18]. Thus, the result with cyproheptadine is only suggestive of a possible effect of antihistamines in migraine treatment.

Sicuteri et al. treated 32 outpatients suffering from migraine with the antihistaminic substance named BC-105, later known as pizotifen. BC-105 also possesses a strong antiserotonin effect. Patients were treated with a maintenance dose of 1.5–2 mg/day (up to 4–5 mg). No predetermined mandatory duration was mentioned and the study was not blinded or placebo controlled. Response to treatment was usually experienced after 10–20 days of treatment. A third of the patients reported absence or significant attenuation of attacks and 40% reported a lower frequency or increased response to acute therapy with ergotamine. Interestingly, BC-105 increased the resilience against migraine attacks induced with trinitroglycerin and reserpine. Side effects were well documented in this study, with 90% of the patients developing an increased appetite. Patients gained 2–5 kg during the first month of treatment. Fatigue and drowsiness was a side effect in 70%. The drug was administered with a starting dose of 0.5 mg twice a day (b.i.d.) increasing to 1 mg b.i.d. after 4–5 days. This was to minimize the initial drowsiness typically experienced during the first period of treatment [19].

Selby et al. studied BC-105 in 40 patients, 11 with migraine with aura, 27 with migraine without aura and 2 with cluster headache. Thirty-five were treated with a dose of 1.5 mg (3 tablets) daily. Two patients received 1 mg and three patients received 2–3 mg/day. The design was uncontrolled without placebo. 42.5% (17 patients) clinically improved with 2 becoming headache free and 15 with a reduction of attack frequency between 25 and 50%. This study reported fewer side effects which the author interpreted as a result of the lesser dose [20].

Another comparative trial investigated three drugs relevant to histamine. Cyproheptadine, BC-105 (pizotifen) and methdilazine, a drug with a four times greater antihistaminic action than promethazine. The patients recruited were not randomly allocated and furthermore had the possibility to shift to another group after 1 month if the current treatment failed. Because of the free movement between the groups, only data for the first month were statistically analyzed. The placebo group of patients was drawn from two other studies conducted simultaneously. Clinical improvement after 1 month was defined as headache free or a reduction in attack frequency of at least 50%. Sixty-one patients received cyproheptadine 4–8 mg three times a day (t.i.d.) of whom 40% improved. Fifty-three patients received 1.5–3 mg of BC-105 t.i.d. with 58% improving and 51 patients received methdilazine 8–16 mg b.i.d. of which 41% improved. All three resulted in a lesser improvement than methysergide, but the compound BC-105 was also significantly better than placebo. Cyproheptadine and methdilazine showed only a positive trend [21].

In a double-blind placebo controlled crossover study, Osterman investigated the effect of Sandomigran® (pizotifen or BC-105), and Divascan®, a serotonin antagonist in migraine prophylaxis. Four patients with migraine with aura and 26 patients with migraine without aura were included. All patients experienced at least 2–3 attacks per month and had a confirmed diagnosis based on then accepted criteria (Ad hoc committee on classification of headache 1962). Three patients discontinued due to adverse events, lack of compliance or unpleasant side effects. Twenty-seven patients were divided into three groups with different treatment sequences of divascan, pizotifen and placebo. Each treatment was given for 8 weeks in a total period of 24 weeks and all three compounds were given in gradually increasing doses using the same amount of capsules. The dose of pizotifen started at 0.5 mg (1 capsule) increasing to a daily maintenance dose of 3 mg (2 capsules, t.i.d.) at day 11. Divascan started at 2.5 mg (1 capsule) increasing to a daily maintenance dose of 15 mg (2 capsules t.i.d.) at day 11. Side effects caused some patients to discontinue the maintenance doses of both drugs. Four patients had only 1.5–2.5 mg/day and 2 patients 10–12.5 mg/day of pizotifen and divascan respectively. The patients kept a headache diary during the trial, with attack frequency, duration and severity as well as the use of attack medication. Severity was rated using an arbitrary 3 point scale and only data from the last 6 weeks of each treatment were analyzed as the first 2 weeks served as wash out period. Treatment with pizotifen gave a significantly lower attack frequency than both placebo and divascan. It also significantly reduced the mean headache index. No significant difference in the duration of attacks was observed. Consumption of ergotamines was significantly higher in the placebo group versus pizotifen and divascan respectively, but not when comparing pizotifen and divascan. In overall evaluation of the treatment divascan did significantly better than placebo but pizotifen had better effect than both placebo and divascan with 70% reporting a good or very good effect. However, when looking at the side effects the authors found that the treatment with pizotifen resulted in significantly more side effects. Most frequent were drowsiness and weight gain (> 1.5 kg) [22].

A multicenter double-blind placebo controlled study in 36 patients in general practice also investigated the effect of pizotifen. All patients had a history of migraine with at least 4 attacks per month. Twenty-four of the patients had failed previous prophylactic therapy. The dose of pizotifen was increased gradually to a daily dose of 1 mg three times a day at day 24 and onwards. The treatment period was 12 weeks and each patient kept a record of frequency and severity of migraine attacks. Twenty-eight patients completed the trial, 14 on pizotifen and 14 on placebo. No statistical analysis was made, but 6 patients reported complete resolution, 6 reported reduced frequency and severity and only 2 patients did not improve clinically. All fourteen patients given placebo failed to show any improvement [23].

Anthony and Lance performed a controlled double blind trial of the H_2_ receptor antagonist cimetidine alone or in combination with the H_1_ receptor antagonist chlorpheniramine as migraine prophylaxis. Twenty-four patients with migraine without aura, and 1 with basilar symptoms received 3 different treatments, each for 1 month during 3 consecutive months. The treatments consisted of 1 month of 200 mg (2 tablets four times a day (q.i.d.)) cimetidine and chlorpheniramine placebo (1 capsule q.i.d.), 1 month of 200 mg (two tablets q.i.d.) cimetidine and chlorpheniramine maleate 4 mg (1 capsule q.i.d) and 1 month of cimetidine placebo (2 tablets q.i.d.) and chlorpheniramine placebo (1 capsule q.i.d.). Patients were randomly allocated to the treatment sequence and kept a record of headache intensity and frequency as well as any side effects. Nineteen patients completed the trial and results were that neither cimetidine in combination with chlorpheniramine nor cimetidine alone proved better than placebo. The authors interpreted the outcome as a result of the intracellular histamine formation by decarboxylation of histidine in multiple tissues, generating headache via other pathways or mechanisms than H_1_ or H_2_ receptors [24]. Another explanation may be a short receptor residence time at the H_1_ receptor by chlorpheniramine (Ki 5–30 nM) [4]. Furthermore, by today’s standards only 1 month of preventative medication is not sufficient to precisely estimate results in migraine prophylaxis. A minimum of 3 months for a phase II and up to 6 months for a phase III study is recommended [25]. Nanda et al. investigated the same drug-placebo combination for 3 months in each group over 9 months combined. Twenty-eight patients with migraine without aura and 6 patients with migraine with aura were included in the trial of which 22 patients began all three periods, but only 16 completed the full 9 months. These patients were allowed to move forward to another treatment group before the 3 months were completed if their headache frequency intensified. The authors failed to show a positive effect on migraine even with a 3 months treatment period. They found the same side effects with drowsiness related to chlorpheniramine and dizziness related to cimetidine [26]. These two studies were blind and placebo controlled and so provide the best evidence. However, using a H_2_ receptor antagonist alone or in combination with H_1_ receptor antagonist showed no significant relief of migraine when used prophylactically.

Cinnarizine, a calcium channel antagonist with H_1_ antihistaminic action and a drug used in Meniere’s disease was tested in migraine prophylaxis. Two open label trials, one with 80 participants and another with 60 tried out cinnarizine in doses up to 75 mg daily in 12 and 14 weeks respectively. Safety and efficacy was measured and both studies generally consider cinnarizine well-tolerated in their participants. Although both groups report a significant reduction of headache frequency the open trial design limits the results to only a hypothetical positive prophylactic effect of cinnarizine [27, 28]. A double blind parallel group study investigated cinnarizine vs. sodium valproate. One hundred four participants were randomized to a daily treatment with either 50 mg cinnarizine (50 participants) or 400 mg valproate (54 participants) for 12 weeks. Dropout rate was ~ 20% for both arms and mainly because of side effects. Valproate proved more effective than cinnarizine but since there was no placebo control efficacy results may be overestimated [29].

A double blind placebo controlled parallel group study of cinnarizine in the prophylaxis of migraine in children was conducted by Ashrafi et al. [30]. Sixty-eight participants aged 5–17 years with migraine with and without aura according to the 2004 IHS criteria for migraine [31] were randomized into 2 groups after 4 weeks of washout. The groups consisted of a treatment group with 50 mg cinnarizine daily (1.5 mg/kg/day for children weighing less than 30 kg) and a placebo group. During a 12 week treatment period participants kept a headache diary and primary outcome measures were frequency, intensity and duration of migraine. Adverse events were too recorded with weight gain and extra pyramidal signs monitored during the trial. Dropout rate was ~ 12% and ~ 5% for the treatment and placebo group, respectively. Cinnarizine significantly reduced headache frequency to < 50% in 60% of the patients compared to only 31.3% in the placebo group.

In conclusion, low level of evidence and conflicting results make it difficult to determine whether or not H_1_ or H_2_ receptor antagonists are effective in migraine treatment. There is a reasonable indication that pizotifen, cinnarizine and also amitriptyline have effect but they have many other effects in addition to being antihistamines. More pure antihistamines such as chlorpheniramine have not shown convincing efficacy. It would be interesting to study the efficacy of pure antihistamines using modern principles of drug trials [25]. The antihistamines used in the trials have primarily an H_1_ antagonistic effect, but also exert antagonism on other receptors, mainly serotonin receptors. The serotonergic effects in migraine is well described elsewhere [32]. Although the complete sites of action remains to be fully described. Serotonin and histamine are both secreted from the mast cells in dura mater [13, 33] and it is likely that the antiserotonin and antihistamine effects potentiate each other, making the results difficult to interpret. Finally the old antihistamines could not be used in high doses because of sedation and weight gain. It might be worth to try high doses of non-sedating modern antihistamines.

### Histamine induction of migraine

The advent of diagnostic criteria for migraine, first unofficial [34] and then internationally accepted ICHD-1 [35] made it possible to further study the relation between histamine induced headache and migraine. Although histamine induction of headache dates back to the 1920s [6] much remained to be elucidated when the modern provocation studies began. The initial hypothesis was that H_1_ and – to a lesser extend – H_2_ receptors on intracranial blood vessels was involved, but the clinical studies [36], experimental studies on cerebral blood flow in human [37] and isolated intra- and extracranial blood vessels [38] found that it was in fact the vasodilatation of the extracerebral vessels that caused the pain.

Krabbe and Olesen investigated a 30 min intravenous infusion of histamine with increasing doses (0.16–0.33-0.66 μg/kg/min.) in 48 participants, 13 healthy volunteers, 10 patients with chronic tension type headache then called chronic muscle contraction headache and 25 patients with migraine without aura, then called common migraine. The objective was to evaluate the sensitivity to histamine in normal subjects and in patients with these headaches and to develop an experimental model of vascular headache. All 48 participants received a continuous infusion of histamine chloride for 30 min*.* 24 of 25 migraineurs developed a throbbing headache, while participants from the two other groups only experienced, at worst, a moderate, pressing type of headache. Adverse events from the histamine infusion consisted mainly of a heating sensation in the face and palpitations. Objectively, all participants developed a flushing of the face, lowering of blood pressure and increased heart rate. No serious adverse events occurred during the study.

In 18 patients who developed headache during the histamine infusion, 0.5 mg/kg of the H_1_ receptor antagonist mepyramine was administered intravenously for the last 2 min of the histamine infusion. Mepyramine greatly reduced or abolished the headache in 15 of 18 migraineurs. In 10 of the patients suffering from migraine who developed a severe throbbing headache, another single blinded crossover study was performed with cimetidine, an H_2_ receptor antagonist. A pretreatment with cimetidine was administered intravenously with a bolus of 3.3 mg/kg, followed by a continuous infusion of 1.66 mg/kg/hour for 45 min finished by the infusion of histamine. The pretreatment with cimetidine slightly but significantly decreased the headache. The only side effect was a metallic taste in the mouth in the majority of the patients [36]. This study proved that histamine induces more or stronger headache in migraine patients than in normal individuals. H_1_ antagonism was effective in aborting the headache and pretreatment with an H_2_ antagonism had some effect.

In a double blind placebo-controlled cross-over study, Lassen et al. investigated whether pretreatment with the H_1_ receptor antagonist mepyramine had an effect on histamine induced headache. In 20 patients with migraine without aura histamine was administered intravenously as an infusion of 0.5 μg/kg/min for 20 min. Prior to this a pretreatment with 0.5 mg/kg mepyramine or placebo (0.9% NaCl) was infused intravenously for 10 min. Data from the first 3 h were documented at the study site and then data were recorded by the patients after discharge for 8 h. Histamine induced an immediate and a delayed headache. Immediate headache was defined as the headache occurring during the first 40 min after start of infusion and the delayed headache was defined as occurring from 40 min to 12 h after start of infusion.

The study was first of its kind describing that a histamine infusion can provoke a delayed genuine migraine attack in migraineurs, as 5 of 10 patients developed an attack fulfilling IHS criteria for migraine without aura. Pretreatment with mepyramine prevented development of both the immediate and the delayed headache [39]. Glyceryl trinitrate (GTN), a nitric oxide donor, is also a potent headache inducer [40] and similar to histamine capable of inducing an immediate headache followed hours later by a delayed migraine-like headache. The headache caused by both substances is biphasic in migraine patients suggesting a shared pathway. However, GTN induced migraine is resistant to H_1_ receptor blockade with mepyramine [41] and histamine could therefore not be the common mediator. The question of nitric oxide being a common mediator of histamine and GTN induced migraine was investigated in two double-blinded and placebo controlled studies, using L-NMMA, a non-selective nitric oxide synthase inhibitor to prevent histamine induced headache in 9 healthy male volunteers [42] and in 12 patients with migraine without aura [43]. Although nitric oxide synthase (NOS) inhibition is effective as a treatment of spontaneous attacks [44, 45], both studies showed no effect on the immediate or delayed headache induced by histamine after pre-treatment with L-NMMA. This underlines the need of further investigation of histamine’s site of action. Because histamine is unable to cross the blood-brain barrier and H_1_ receptors are present in the endothelium of cerebral arteries, migraine attacks could have an origin in the walls of intracerebral arteries and not in the brain itself [41].

Histamine inhalation was investigated by Lassen et al. in 12 patients with migraine without aura, 2 patients with migraine with aura and 1 patient with migraine both with and without aura. They were matched with 15 healthy volunteers in a double blind study. All participants underwent 2 min inhalation periods with 2-fold increase in dose of histamine for every period. The objectives were to evaluate whether histamine inhalation could provoke a headache, whether it showed dose-dependency, whether induced attacks were genuine migraine attacks and whether attacks were induced with the same frequency in both groups. Immediate and delayed headache were reported in both groups with no significant difference, but only migraineurs (6 of 15) developed a headache fulfilling the IHS criteria for migraine without aura. Development of migraine after inhalation had a positive predictive value of 1.0, but the sensitivity was poor. The clinical signs and symptoms other than headache were flushing, heat sensation, palpitation, coughing, dizziness and dysphonia, none of which were severe [46].

Histamine plasma levels in migraine patients have been reported elevated compared with controls both ictally and interictally [47] while another study reported no change during attacks but significant difference between migraineurs and controls between attacks [48]. Latest unpublished results from a biomarker study in patients with migraine without aura using mass spectrometry found that histamine levels both during and outside of attacks were below detection limit and we will not review this further.

### H_3_ and H_4_ receptor pharmacology and possible involvement in migraine

**Fig. 2:**
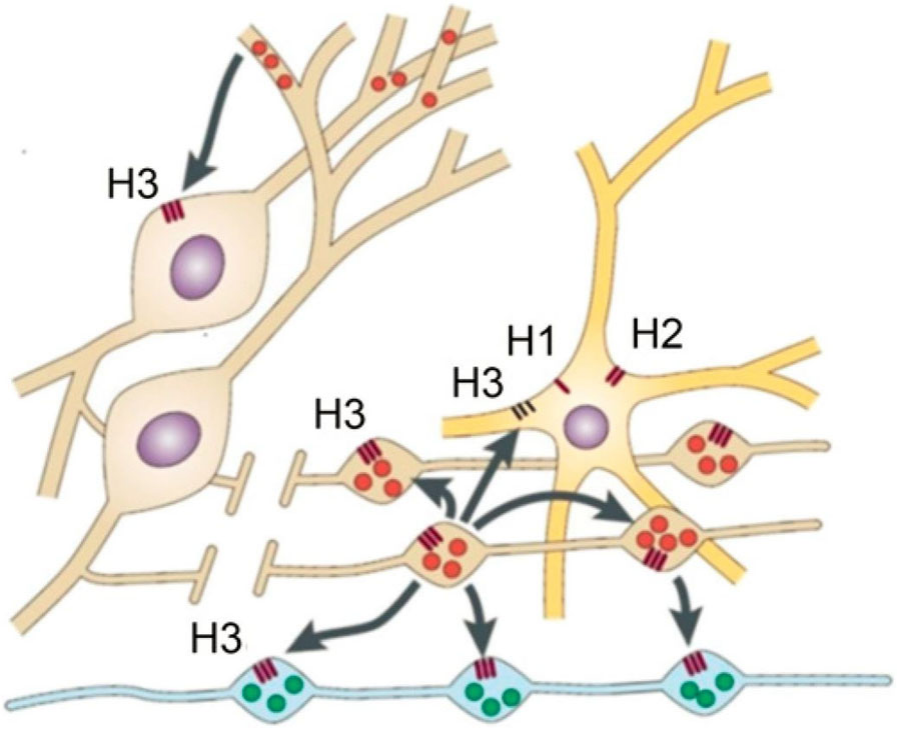
Neuronal H3 receptors are located on both histaminergic (brown) and non-histaminergic neurons (arrows). On histaminergic neurons H3 acts as an autoreceptor inhibiting the release of histamine itself. The figure shows the complex distribution of H_3_ receptors that emphasizes the challenge of predicting the effects mediated by H_3_ receptors. Reprinted from (12) Copyright© 2015 with permission from ASPET, Pharmacol Rev.

A third member of the histamine receptor family was discovered pharmacologically by Arrang in 1983 when he showed that histamine inhibited its own release from depolarized slices of rat cerebral cortex. The receptor responsible was identified as a presynaptic inhibitory receptor – an autoreceptor – independent of other neurotransmitter antagonists and with a different ligand profile. He concluded that a novel class of histamine receptors was discovered and proposed the name H_3_ [49]. The histamine H_3_ receptor cDNA was cloned by Lovenberg and colleagues in 1999 and they described an extensive expression in the rat CNS especially in the caudate nucleus, thalamus and cortex [50]. Apart from the receptor’s full length polypeptide approximately 20 human isoforms have been described [3]. Studies of rat, guinea pig and human brains contribute to the knowledge of the H_3_ receptor’s expression in mammalian CNS. The H_3_ receptor has been mapped by various methods such as autoradiography and later by in situ hybridization and reverse transcription polymerase chain reaction of H_3_ receptor mRNA. Countless studies report expression of the H_3_ receptor in the brain of rodents, guinea pigs, monkeys and humans. Thus there is good evidence that histamine H_3_ receptor is present in many important brain structures e.g. cortex, cerebellum, dorsal thalamus, hypothalamus, amygdala, hippocampus, striatum and basal ganglia. The receptor is primarily located as a presynaptic autoreceptor on histaminergic neurons (on dendrites, axons and somata), which are localized in the tuberomamillary nucleus in the posterior hypothalamus of rodents and humans, or as a heteroreceptor in non-histaminergic neurons. There is some evidence for a postsynaptic H_3_ receptor in the CNS as well as a location in the PNS (Fig. 2) [3, 51, 52].

**Fig. 3:**
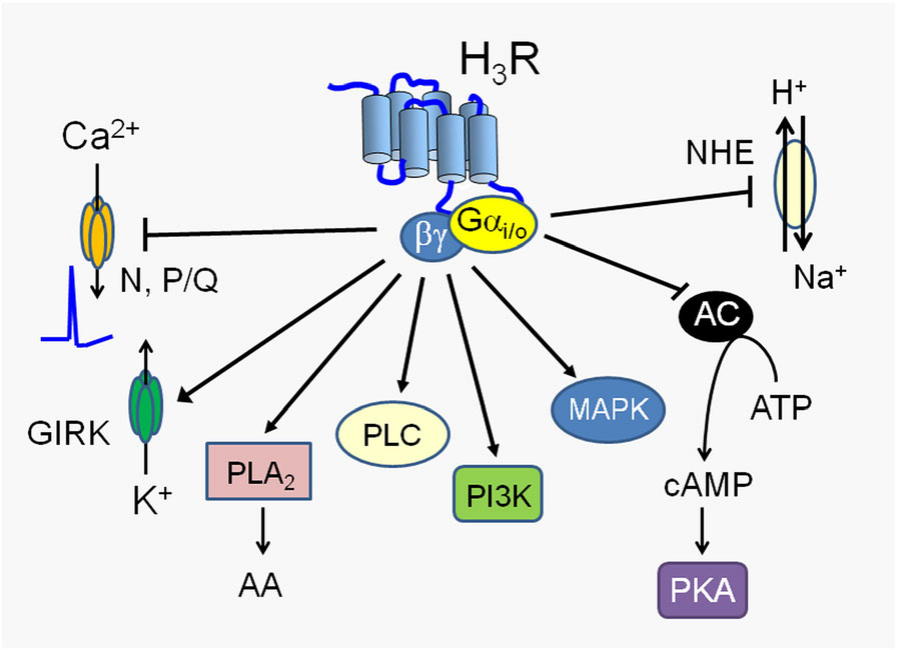
Downstream signaling from the H3 receptor. Opposite to the H_2_ receptor, adenylate cyclase is inhibited by the H_3_ receptor. Reprinted from (13) Copyright© 2016 with permission from ASPET, Mol Pharmacol

The H_3_ receptor alone has been reviewed recently [52] and alongside the H_1_, H_2_ and H_4_ receptors [3]. The histamine H_3_ receptor has multiple signal transduction mechanisms. The receptor couples to G_i/o_ protein leading to inhibition of adenylate cyclase (AC) and a reduction of cytosolic cAMP (the opposite action of the H_2_ receptor which stimulates AC) is one of the main signaling mechanisms (Fig. 3). The H_3_ receptor also signals by activation of mitogen-activated protein kinase (MAPK), by the Akt/PKB signaling pathway and via activation of phospholipase C (PLC) leading to an increase in cytosolic calcium with the latter leading to an inhibition of histamine synthesis and release, hence the auto-receptor function [3].

**Fig. 4:**
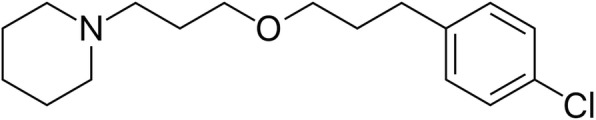
Structural formula of pitolisant

The numerous functions of the histamine H_3_ receptor reflect its widespread localization in the central and peripheral nervous systems as well as interactions with - and influence by - other neurotransmitters and neuropeptides. Many functions are still hypothetical or controversial, particularly the effects in vivo. In general, the receptor mediates the function of histamine itself in the nervous system. The autoreceptor inhibits the release of histamine from histaminergic neurons and this receptor has a significant constitutive activity which refers to the ability of signaling independent of agonist action. The presynaptically located H_3_ receptor regulates the release of histamine and other neurotransmitters (serotonin, GABA, glutamate, noradrenaline and possibly acetylcholine and dopamine) and neuropeptides in the CNS and PNS (Fig. 4). This links the histamine H_3_ receptor to several neurological and psychiatric disorders with H_3_ agonists being potential treatments in Alzheimer’s and Parkinson’s disease, schizophrenia, sleep disorders and addiction [52]. H_3_ agonists might also perhaps be effective in migraine.

The role of histamine in neurogenic inflammation has been reviewed by Rosa and Fantozzi who described an interaction of mast cells and afferent nerve fibers. Mast cell degranulation is stimulated by release of neuropeptides (e.g. CGRP, substance P and VIP) from the nerve endings [16, 53] and is inhibited by histamine via the H_3_ receptor [54]. The degranulation of mast cells promotes an inflammatory response including neurogenic inflammation. This leads to a release of inflammatory mediators by afferent neurons which in turn stimulate other mast cells to degranulate and thereby sustain and prolong the inflammation. There is evidence that mast cell degranulation activates meningeal nociceptors and it is possible that mast cell degranulation has a role in migraine [13, 33, 53, 55]. A potential therapeutic target would be a receptor with a stabilizing function on mast cells preventing or minimizing the degranulation and secondly the neurogenic inflammation. Another possible target is a receptor located on the nerve terminals inhibiting the release of neuropeptides. The H_3_ receptor agonist R^α^-methylhistamine inhibited plasma protein extravasation following capsaicin or electrical stimulation of the trigeminal nerve [56]. This suggests that neuropeptide release from sensory C fibers in the rat meninges is at least partially controlled by H_3_ receptors. Better knowledge of these mechanisms in neurogenic inflammation could create a possibility of intervening with receptor specific drugs.

Histamine reduces nociceptive transmission when injected into the cerebrospinal fluid or microinjected in brain structures. Central H_1_ and H_2_ receptors are thought to mediate this effect, but theoretically H_3_ receptors could also be involved. An antagonist effect on the H_3_ receptor leads to an increase of brain histamine and several basic studies of acute and chronic pain tests have investigated H_3_ antagonism as a modifier of nociceptive thresholds. Investigation of thioperamide, an H_3_ receptor inverse agonist, reduces nociception in rodents, but the effect is limited and shows a biphasic response with decreasing effects at highest doses [57].

A single research group investigated doses of 1–10 ng histamine injected subcutaneously twice a week as migraine treatment [58] and subsequently compared it with topiramate [59] in patients with refractory migraine with positive results. Their hypothesis was that histamine acts as an agonist on H_3_ receptors on mast cells thereby reducing the neurogenic inflammation. The group continued with an investigation of the H_3_ agonist N^α^-methylhistamine in migraine prophylaxis. They published their results from phase I and II studies with 30 healthy volunteers and 17 patients with migraine without aura and 1 patient with migraine with aura. Doses of 1–3 ng subcutaneous twice a week proved effective in migraine patients with a reduction of frequency, intensity and duration of the attacks. Adverse effects were intense headache with doses above 10 ng and 4 ng in healthy volunteers and migraine patients respectively. The authors attributed this to an increased H_1_ agonistic effect [60]. A double blind placebo controlled phase III study was performed and results were published in 2006. Sixty patients with migraine (50 migraine without aura and 10 migraine with aura) were included. Two from the placebo group and 3 patients from the N^α^ group left the study and 55 patients completed the 12 week period. The study confirmed the results seen in the phase II trial along with lower intake of rescue analgesics in the N^α^-group vs. the placebo group and without any adverse events. The hypothesis in the study was that low doses of a H_3_ agonist modulates the interaction between mast cells and C-fiber endings and by reducing histamine release lowering the neurogenic inflammation [61]. The doses used in these studies are unusually low making it difficult to understand the mechanism of action or to even accept the results of such minute doses. Furthermore, N^α^-methylhistamine does not cross the blood brain barrier. Apparently, no filing for registration of N^α^-methylhistamine has been done, so industry is not convinced of these results.

The only H_3_ receptor agent approved for medical use is pitolisant, an H_3_ inverse agonist. Pitolisant has recently been discovered to be an efficient treatment of narcolepsy and marketed by Bioprojet Pharma as Wakix. The mechanism behind is increased histamine in the brain leading to an increased wakefulness. One of the pivotal studies described headache as an adverse event in 35% of the participants [62]. In a review by Schwartz of the first clinical trials with pitolisant in narcolepsy and Parkinson’s disease, he also describes headache as an adverse event of pitolisant. Overall in these studies 9.7% developed headache on pitolisant compared to 2.9% on placebo [63]. Pitolisant readily crosses the blood brain barrier [3] but histamine is known to lack this ability. It remains unclear if this headache observed in the pitolisant trials is due to central or peripheral mechanisms but it is interesting that a drug which promotes histamine release is linked to an increased susceptibility of headache.

The discovery of the histamine H_4_ receptor was reported by several independent research groups around the turn of the millennium. The H_4_ receptor has ~ 40% homology with the H_3_ receptor. Two additional inactive isoforms have been identified [3]. The H_4_ receptor is a coupled to G_i_/G_o_ protein and uses intracellular Ca^2+^ as the primary second messenger. In recombinant systems cAMP increase or decrease has been coupled to an agonist response and inverse agonist response, respectively, indicating mediation by adenylate cyclase. There are probably additional signaling mechanisms and supporting evidence is emerging. The localization of the H_4_ receptor is complicated by deficiency of validated H_4_ selective antibodies to identify the H_4_ receptor expression. Consensus is that H_4_ receptors are present in the bone marrow and hematopoietic cells. H_4_ receptor expression was also shown in sensory neurons innervating skin in mice but there is no evidence for its presence in human sensory neurons yet. Pharmacological methods have shown that the H_4_ receptor is expressed on mast cells [3] and there is some evidence that microglial cells express H_4_ receptors [64].

The literature describes an immune modulating function of the H_4_ receptor with an involvement in inflammatory responses. It mediates chemotaxis and is involved in cytokine release from various immune cells. Coruzzi et al. suggested anti-inflammatory and antinociceptive effects of H_4_ antagonists in a rat model [65], a characteristic shared with the H_3_ receptor [3]. The H_4_ receptor has not yet been associated with migraine and further description of its localization and function is needed.

## Conclusions and future perspectives

Histamine receptors H_1_, H_2_, H_3_ and H_4_ are widespread throughout the body but there is limited knowledge about the H_4_ receptor. H_3_ is an autoreceptor, inhibiting release of histamine where H_1_ and H_2_ have pure agonistic effects and have effect on multiple sites. Modern provocation studies with histamine have increased the interest in the histaminergic system in relation to migraine because histamine proved to be an effective migraine provoking agent. Studies of the effect of antihistamines in migraine are limited and of poor quality. Thus, there is little support for H_1_ or H_2_ antihistamines as preventive migraine medication. In addition, there are undesirable adverse effects. Still, non-sedating H_1_ antihistamines need to be appropriately tested. This has not been done and it might provide valuable information of histamine’s site of action and it’s relation to migraine. There are positive reports about the H_3_ receptor agonist N^α^-methylhistamine as migraine treatment, but the use of almost homeopathic doses makes the results less credible. Additional exploration of the histaminergic system, peripheral and central, could reveal further goals for migraine therapy.

## Abbreviations

AC: Adenylate cyclase
B.i.d.: Two times a day
CGRP: Calcitonin gene-related peptide
GTN: Glyceryl trinitrate
MAPK: Mitogen-activated protein kinase
NOS: Nitric oxide synthase
PLC: Phospholipase C
Q.i.d.: Four times a day
T.i.d.: Three times a day
VIP: Vasoactive intestinal peptide
